# Attributable causes of colorectal cancer in China

**DOI:** 10.1186/s12885-017-3968-z

**Published:** 2018-01-05

**Authors:** Meng-Jia Gu, Qiu-Chi Huang, Cheng-Zhen Bao, Ying-Jun Li, Xiao-Qin Li, Ding Ye, Zhen-Hua Ye, Kun Chen, Jian-Bing Wang

**Affiliations:** 1 Department of Epidemiology and Health Statistics, Zhejiang University School of Public Health, 866 Yuhangtang Road, Hangzhou, 310058 China; 2 Sir Run Run Shaw Hospital, School of Medicine, Zhejiang University, Hangzhou, 310058 China; 3 School infirmary of Zhejiang University, Hangzhou, 310058 China

**Keywords:** Colorectal cancer, Risk factors, Population attributable fraction, China

## Abstract

**Background:**

Colorectal cancer is the 4th common cancer in China. Most colorectal cancers are due to modifiable lifestyle factors, but few studies have provided a systematic evidence-based assessment of the burden of colorectal cancer incidence and mortality attributable to the known risk factors in China.

**Methods:**

We estimated the population attributable faction (PAF) for each selected risk factor in China, based on the prevalence of exposure around 2000 and relative risks from cohort studies and meta-analyses.

**Results:**

Among 245,000 new cases and 139,000 deaths of colorectal cancer in China in 2012, we found that 115,578 incident cases and 63,102 deaths of colorectal cancer were attributable to smoking, alcohol drinking, overweight and obesity, physical inactivity and dietary factors. Low vegetable intake was the main risk factor for colorectal cancer with a PAF of 17.9%. Physical inactivity was responsible for 8.9% of colorectal cancer incidence and mortality. The remaining factors, including high red and processed meat intake, low fruit intake, alcohol drinking, overweight/obesity and smoking, accounted for 8.6%, 6.4%, 5.4%, 5.3% and 4.9% of colorectal cancer, respectively. Overall, 45.5% of colorectal cancer incidence and mortality were attributable to the joint effects of these seven risk factors.

**Conclusions:**

Tobacco smoking, alcohol drinking, overweight or obesity, physical inactivity, low vegetable intake, low fruit intake, and high red and processed meat intake were responsible for nearly 46% of colorectal cancer incidence and mortality in China in 2012. Our findings could provide a basis for developing guidelines of colorectal cancer prevention and control in China.

## Background

Colorectal cancer has ranked the third most common cancer and the fourth most common cancer cause of death worldwide, and almost 1.4 million new cases and 694,000 deaths occurred in 2012 [1]. The incidence rate is low among people aged less than 50 years, but getting strong increase with age [2]. Colorectal cancer rates are higher in some developed countries such as the Czech Republic, Japan, Australia, the majority of Western Europe and North America, which may result from the “Westernization” (such as obesity and physical inactivity) [3]. According to the WHO data, China suffered 245,000 new cases and 139,000 deaths of colorectal cancer in 2012, which made it the fifth most common cancer in man and the fourth in women.

Recently, it is believed that no single risk factor could be responsible for risk of colorectal cancer [4]. Individuals who had higher education level or were non-Hispanic white suffered lower risk of colorectal cancer mortality [5]. Over the past few decades, a number of risk factors for colorectal cancer have been identified, including: family history of colorectal cancer [6], inflammatory bowel disease [7], diabetes [8], obesity [9], excessive alcohol consumption [10], high consumption of red and processed meat [11], low vegetable and fruit intake [12] and tobacco smoking [13].

The attributable causes of colorectal cancer have been reported in western populations and results remained inconsistent [14–17]. A study from Japan has estimated that 33.6% and 31.7% colon cancer cases and deaths were attributable to the selected risk factors in 2005, including smoking, alcohol drinking, body fatness and physical inactivity [17]. A previous study in China [18] aimed to estimate the contribution of known causes of cancer in 2005, including smoking, alcohol drinking, chronic infection, nutritional factors, overweight and obesity, physical inactivity, occupational factors and hormonal factors, has showed that about 14.6% of colon cancer deaths were attributable to alcohol drinking, overweight and obesity and physical inactivity. However, previous studies of comprehensive assessment on the colorectal cancer burden in China have been limited. Moreover, the socio-economic status and lifestyle patterns have been changing in China due to the rapid development of economy.

Herein, we comprehensively evaluate the fraction of colorectal cancer cases and deaths attributable to demonstrated risk factors in China in 2012. Our results would be informative for colorectal cancer control and prevention in China and other countries with similar profiles.

## Methods

### Overview

Our study aimed to estimate the numbers and proportions [Population Attributable Fractions (PAFs)] of colorectal cancer incidence and mortality in China in 2012 that could be attributable to the documented risk factors. PAF is defined as the fraction of cancer that can be attributable to a risk factor. For avoidable risk factors, PAF is the proportion of cancer that can be avoided by modifying or removing the exposure of certain causal factors.

Since several studies have been published about certain exposures in China [19–21], we focused on the joint effects of these risk factors on colorectal cancer and comparing these joint effects with those found in other similar studies. Two independent investigators were involved in performing the literature searches, extracting the data and supervising statistical analyses.

### Colorectal cancer incidence and mortality

Colorectal cancer incidence and mortality data in China were derived from the Globocan project in IARC in 2012. The specific data sources and methods are described in the website (http://globocan.iarc.fr/Pages/DataSource_and_methods.aspx) [22]. Briefly, data on colorectal cancer mortality were collected from the registration system in China, while the incidence data was estimated from the known cancer deaths and Mortality and Incidence (M/I) ratio that was calculated using Poisson regression adjusted for age, gender and regional sites. In China, National Center of Cancer Register was established in 2002. In 2012, there were 222 population-based cancer registries in China, which covered approximately 15% of the Chinese national population.

Overall, the most recent cancer facts showed that 139,000 people died from colorectal cancer in China in 2012, with 79,000 in men and 60,000 in women separately. The incident cases of colorectal cancer was 253,000, including 147,000 in men and 107,000 in women.

### Selection of risk factors

The risk factors included in this study were those have evidence for a causal association with colorectal cancer according to two reports (Table 5 in Appendix 1). One was the World Cancer Report 2008 from the International Agency for Research on Cancer (IARC) [23], which identified dietary risk factors (high red and processed meats intake, and low vegetable and fruit intake), cigarette smoking, alcohol drinking, obesity, and physical inactivity. The second was the Colorectal Cancer 2011 Report from the Continuous Update Project (CUP) of the World Cancer Research Fund International (WCRF)that identified red or processed meats, alcohol drinking, overweight/obesity and physical inactivity as convincing risk factors, and low vegetable and fruit intake as probable risk factors for colorectal cancer [24]. We included all the convincing and probable risk factors in our study to estimate the joint effect of these exposures on colorectal cancer incidence and mortality.

### Prevalence of exposure data

The current health effects of risk factors are a result of the accumulative patterns of past exposure to these risk factors, we estimated an average induction time of 10–15 years for risk factors and colorectal cancer [14, 15] and therefore obtained exposure data from 1997 to 2002. Prevalence of exposure data was extracted from nationally representative studies in China (Table 6 in Appendix 1).

#### Prevalence of tobacco smoking in China

Tobacco smoking prevalence was abstracted from the results of a cross-sectional study in 2002 on smoking and passive smoking status in China [25]. Briefly, 145 disease surveillance points (DSPs) in 30 provinces throughout the country were selected by multi-stage random sampling method. A total of 16,056 valid records were included in the final analysis. Smokers were defined as persons who smoked at least 100 cigarettes or had ever smoked for at least 6 months. Current smokers were smoking cigarettes at the time of survey. The overall prevalence of tobacco smoking was 57.4% in men and 2.60% in women.

#### Prevalence of alcohol drinking in China

Data on alcohol drinking prevalence was obtained from the 2002 National Nutrition Survey of China [26],and the specific methods of this study have been described elsewhere [27]. Briefly, the survey covered more than 240 thousand persons aged over 15 years in 31 provinces, autonomous regions and municipalities, including Hong Kong, Macao and Taiwan using the multi-stage stratified cluster random sampling method. Alcohol consumption was defined as drinking alcohol on at least 12 occasions during the past 12 months. We did not take into account the type of alcohol (beer, wine, distilled spirit) or drinking patterns (regular vs. binge drinking) due to lack of this information. Overall, the prevalence of alcohol drinking was 39.6% in men and 4.5% in women, respectively.

#### Prevalence of physical inactivity in China

We obtained the prevalence of physical inactivity from the International Collaborative Study of Cardiovascular Disease in Asia (InterASIA study), which conducted in the Chinese general population aged 35 to 74 years between 2000 and 2001 [28].A total of 14,933 persons completed the questionnaire and provided the nation-based prevalence of physical inactivity. Participants were asked to the number of hours per day that they devoted to vigorous or moderate or light activity, and physical inactivity was defined as participating in less than 30 min of moderate or vigorous activity per day. Results showed that 30.4% in men and 36.8% in women were physical inactivity.

#### Prevalence of overweight and obesity

Prevalence data on overweight and obesity were derived from the Chinese health and nutrition survey (CHNS) in 2000, which was conducted by the Chinese Academy of Preventive Medicine and the University of North Caroline Population Center [29]. The 2000 CHNS covered 15,648 participants from 9 representative provinces (54 counties) that varied in economic development, geography circumstances, public resources and health status.

Body height and weight data were obtained from the physical examination records of 2000 CHNS. We used body mass index (BMI) to evaluate the health effects of body weight, which can be calculated as the weight divided by the square of the height (kg/m^2^). For international comparisons, WHO recommends the following criteria: BMI at 25.0–29.9 kg/m^2^ as “overweight” and 30 kg/m^2^ or higher as “obesity”. The estimated overall prevalence of overweight in 2000 was 15.03% in men and 16.97% in women, while the prevalence of obesity was 2.49% in men and 3.41% in women, respectively.

#### Prevalence of dietary factors

Prevalence data on dietary factors were also derived from the Chinese Health and Nutrition Survey in 2000 that was described earlier. Dietary factors including intake of vegetable and fruit, and intake of red and processed meats, were derived from the household survey of 2000 CHNS. These factors were achieved as continuous variables and defined as the mean per capita dietary intake, measured in Liang (equals to 50 g) per day using a 24-h recall method. In our study, we categorized intake of vegetable and fruit, red and processed meats in quintiles, stratified by regions (urban and rural) and genders.

The prevalence of dietary factors varied from urban to rural areas and between genders in China. Table 1 presents the distribution of fruit, vegetable and red/processed meat intake (g/d) in 2000 in China. In urban areas, the highest quintile of vegetable intake was over 441.7 g/d in men and 400 g/d in women, respectively. For fruit intake, the highest quintile was over 183.3 g/d in men and over 200 g/d in women. The highest quintile of red and processed meat intake was the same with fruit in men and over 150 g/d in women. In rural areas, the highest quintiles of these three dietary factors intake were slightly lower than those in urban areas except for the highest quintile of fruit in women.

**Table 1:** Distribution of fruit, vegetable and red/processed meat intake (g/d) in 2000 in China

Quintile	Urban men			Urban women			Rural men			Rural women		
	Vegetable	Fruit	Red/processed meat	Vegetable	Fruit	Red/processed meat	Vegetable	Fruit	Red/processed meat	Vegetable	Fruit	Red/processed meat
1	<166.7	<50.0	<50.0	<150.0	<50.0	<33.3	<155.0	<33.3	<33.3	<150.0	<33.3	<33.3
2	166.7–249.9	50.0–83.2	50.0–83.2	150.0–224.9	50.0–83.2	33.3–66.6	155.0–233.2	33.3–66.6	33.3–59.9	150.0–219.9	33.3–66.6	33.3–49.9
3	250.0–324.9	83.3–116.6	83.3–119.9	225.0–299.9	83.3–116.6	66.7–99.9	233.3–316.6	66.7–88.2	60.0–86.6	220.0–299.9	66.7–99.9	50.0–83.2
4	325.0–441.6	116.7–183.2	120.0–183.2	300.0–399.9	116.7–199.9	100.0–149.9	316.7–433.2	88.3–176.6	86.7–136.6	300.0–399.9	100.0–233.2	83.3–116.6
5	≥441.7	≥183.3	≥183.3	≥400.0	≥200.0	≥150.0	≥433.3	≥176.7	≥136.7	≥400.0	≥233.3	≥116.7

Data were extracted from the Chinese health and nutrition survey (CHNS) in 2000 [29]

### Relative risk (RR) data

Data on the RRs of different risk factors and risk of colorectal cancer in this study was derived from epidemiologic studies through a systematic search of publications, including: PubMed, Web of Science, websites, and China National Knowledge Infrastructure (CNKI). The selection criteria for RRs should include relative risks or odds ratios and the corresponding 95% confidence intervals (CIs). Language was limited to English or Chinese only. The highest priority was given to those meta-analyses or large-scale cohort studies in the Chinese population. When such studies were not available, we used meta-analyses of other Asian or non-Asian populations. Furthermore, if RRs for men and women were not available separately, we assumed the RRs for both men and women were equal. All RRs used in our study were statistically significant.

RR for tobacco smoking was from a meta-analysis of 22 cohort studies in Asian and other populations [30], and RR for alcohol drinking was derived from a population-based cohort study in the Chinese population [31]. However, RRs for overweight and obesity were obtained from the Asia-Pacific Cohort Studies Collaboration in Asian and other populations [32], and RR for physical inactivity was from a meta-analysis included 53 studies in Asian and other populations [33].

RRs for vegetable and fruit [12] and red or processed meat intake [11] were abstracted from two large studies of meta-analyses on the dose-response association with colorectal cancer incidence. These two studies both estimated the RRs for every 100 g/day increase. The RRs calculated in our study were first transformed onto a log scale and divided by 100 to give the log RR/g per day, then multiplied by the lower intake of every quintile. Finally, the RRs in other quintiles was divided by that in quintile 5 (fruit or vegetable) or in quintile 1(red or processed meat) to get the final estimates. The RR in quintile 5 (fruit or vegetable) or in quintile 1(red or processed meat) was assumed to be equal to 1.

Table 2 shows RRs and 95% CIs for the selected risk factors and risk of colorectal cancer. RR for smoking (current vs. never) from the meta-analysis was 1.16 (95% CI, 1.09, 1.24) and as compared with non-drinkers, the combined RR for drinkers was 1.24 (95% CI, 1.01, 1.54) for both sexes. For overweight and obesity, the RRs were 1.25(95% CI, 1.07, 1.45) and 1.57(95% CI, 1.23, 2.00) for both men and women. For physical inactivity, the RRs were 1.32 for men and 1.27 for women, respectively. For the lowest quintile of vegetable intake, RRs were similar among urban and rural men and women (1.52 vs. 1.46 for urban men and women, 1.50 vs. 1.46 for rural men and women). For the lowest quintile of fruit intake, the RRs were 1.12 and 1.13 among urban men and women, and the corresponding figures were 1.12 and 1.16 among rural men and women. When considering the highest intake of red and processed meat, the RRs were 1.27 among urban men and 1.22 among urban women, and the corresponding figures were 1.20 in rural men and 1.17 in rural women, respectively.

**Table 2:** Relative risks (RRs) and 95% confidence intervals (CIs) between selected risk factors and risk of colorectal cancer

Risk factors	RR (95% CI)			Sources
	Men	Women	Design	
Smoking	1.16 (1.09–1.24)	1.16 (1.09–1.24)	Meta-analysis	30
Alcohol drinking	1.24 (1.01–1.54)	1.24 (1.01–1.54)	Cohort study	31
Overweight	1.25 (1.07–1.45)	1.25 (1.07–1.45)	Cohort study	32
Obesity	1.57 (1.23–2.00)	1.57 (1.23–2.00)		
Physical inactivity	1.32 (1.23–1.41)	1.27 (1.17–1.41)	Meta-analysis	33
Vegetable intake^a^: Urban				12
Quintile 5	1.00	1.00		
Quintile 4	1.12 (1.04–1.19)	1.10 (1.03–1.16)		
Quintile 3	1.20 (1.06–1.34)	1.18 (1.05–1.30)		
Quintile 2	1.30 (1.09–1.51)	1.27 (1.08–1.46)		
Quintile 1	1.52 (1.14–1.95)	1.46 (1.13–1.83)		
Vegetable intake^a^: rural			Meta-analysis	12
Quintile 5	1.00	1.00		
Quintile 4	1.12 (1.04–1.19)	1.10 (1.03–1.16)		
Quintile 3	1.21 (1.06–1.35)	1.19 (1.06–1.31)		
Quintile 2	1.30 (1.09–1.52)	1.27 (1.08–1.46)		
Quintile 1	1.50 (1.14–1.92)	1.46 (1.13–1.83)		
Fruit intake^a^: urban			Meta-analysis	12
Quintile 5	1.00	1.00		
Quintile 4	1.04 (1.01–1.07)	1.05 (1.02–1.09)		
Quintile 3	1.06 (1.02–1.11)	1.07 (1.02–1.13)		
Quintile 2	1.09 (1.03–1.15)	1.10 (1.03–1.17)		
Quintile 1	1.12 (1.04–1.21)	1.13 (1.04–1.23)		
Fruit intake^a^: rural			Meta-analysis	12
Quintile 5	1.00	1.00		
Quintile 4	1.06 (1.02–1.10)	1.09 (1.03–1.15)		
Quintile 3	1.07 (1.02–1.12)	1.11 (1.03–1.19)		
Quintile 2	1.09 (1.03–1.16)	1.13 (1.04–1.23)		
Quintile 1	1.12 (1.04–1.20)	1.16 (1.05–1.28)		
Red and processed meat^a^: urban			Meta-analysis	11
Quintile 1	1.00	1.00		
Quintile 2	1.07 (1.02–1.11)	1.04 (1.01–1.07)		
Quintile 3	1.12 (1.03–1.20)	1.09 (1.03–1.15)		
Quintile 4	1.17 (1.05–1.29)	1.14 (1.04–1.24)		
Quintile 5	1.27 (1.07–1.48)	1.22 (1.06–1.38)		
Red and processed meat^a^: rural			Meta-analysis	11
Quintile 1	1.00	1.00		
Quintile 2	1.04 (1.01–1.07)	1.04 (1.01–1.07)		
Quintile 3	1.08 (1.02–1.14)	1.07 (1.02–1.11)		
Quintile 4	1.12 (1.03–1.21)	1.12 (1.03–1.20)		
Quintile 5	1.20 (1.06–1.34)	1.17 (1.05–1.29)		

^a^RRs were first transformed onto a log scale and divided by 100 to give the log RR/g per day, then multiplied by the lower intake in each quintile, finally, the RRs in other quintiles were divided by that in quintile 5 (vegetable and fruit) or quintile 1 (red and processed meat) to get the final RRs, and the RR in quintile 5 (vegetable and fruit) or quintile 1 (red and processed meat) was assumed to be equal to 1

### Statistical analysis

PAF was estimated based on the RR of cancer associated with exposure to a particular risk factor and the prevalence of exposure to the risk factor in a general population (P). The colorectal cancer-specific PAF for different exposures for 2012 was calculated using Levin’s formula [34]:

$$ \mathrm{PAF}=\frac{\mathrm{P}\times \left(\mathrm{RR}-1\right)}{\left[\mathrm{P}\times \left(\mathrm{RR}-1\right)\right]+1} $$

PAFs for low intake of vegetable and fruit and high intake of red or processed meat were calculated by a shift of all to the top quintile, which is a full shift, by the following formula:

$$ \mathrm{PAF}=\frac{\sum \limits_{i=1}^n{P}_i\left({RR}_i-1\right)}{\sum \limits_{i=1}^n{P}_i\left({RR}_i-1\right)+1} $$

RR_i_ was the relative risk at quintile i (*i* = 1, 2, 3, 4, 5).

P_i_ was the prevalence of quintile i in a full shift, which was 20%.

Combined PAF for risk factors in our study including smoking, alcohol drinking, overweight/obesity, physical inactivity, low vegetable and fruit intake, and high red and processed meat intake can be estimated by the following formula [35]:

$$ \mathrm{PAF}=1-\left(1-\mathrm{PA}{\mathrm{F}}_1\right)\times \left(1-\mathrm{PA}{\mathrm{F}}_2\right)\times \left(1-\mathrm{PA}{\mathrm{F}}_3\right)\times \left(1-\mathrm{PA}{\mathrm{F}}_4\right)\times \left(1-\mathrm{PA}{\mathrm{F}}_5\right)\times \left(1-\mathrm{PA}{\mathrm{F}}_6\right)\times \left(1-\mathrm{PA}{\mathrm{F}}_7\right) $$

Where PAF_1_ is the PAF for exposure to tobacco smoking, PAF_2_ is the PAF for exposure to alcohol drinking, PAF_3_ is the PAF for exposure to overweight and obesity, PAF_4_ is the PAF for exposure to physical inactivity, PAF_5_ and PAF_6_ are the PAFs for exposure to low vegetable and fruit intake, and  PAF_7_ is the PAF for exposure to high red and processed meat intake.

In addition, considering the uncertainty in the estimation of PAF resulting from RR and prevalence of exposure, a delta method was used in the standard formula for estimating the 95% CI of PAF [36].

## Results

The PAFs of colorectal cancer incidence and mortality are listed in Table 3. Tobacco smoking was responsible for 6636 deaths and 12,348 incident cases of colorectal cancer in men with a PAF of 8.4% (95% CI, 4.9%, 12.1%); and for women, the corresponding deaths and cases were only 240 and 428 with a PAF of 0.4% (95% CI, 0.2%, 0.6%). The fraction of colorectal cancer incidence and mortality caused by alcohol drinking was 8.7% for men (95% CI, 0.4%, 17.6%) and 1.1% for women (95% CI, 0.0%, 2.4%). The corresponding figures were 6873 deaths and 12,789 incident cases in men and 660 deaths and 1177 incident cases in women. The estimates of PAF for overweight and obesity was 4.9% (95% CI, 1.6%, 8.5%) in men and 5.8% (95% CI, 1.9%, 9.9%) in women. Furthermore, physical inactivity was responsible for 8.9% of colorectal cancer incidence and mortality in men (95% CI, 6.5%, 11.1%) and 9.0% (95% CI, 5.9%, 13.1%) in women. When increasing vegetable and fruit consumption, we could save 33,850 of colorectal cancer deaths and 61,895 incident colorectal cancer cases (PAF = 17.9% for low vegetable intake and 6.4% for low fruit intake). High red and processed meat intake was responsible for 8.6% (2.5%, 13.9%) of colorectal cancer incidence and mortality (PAF = 9.1% for men and 7.9% for women). Overall, we estimated that 63,102 (PAF = 45.5%; 95% CI: 18.1%, 63.8%) of the total colorectal cancer deaths, including 39,342 of the deaths in men and 23,760 deaths in women, and 115,578 incident colorectal cancer cases (73,206 in men and 42,372 in women) in 2012 were attributable to the combined effects of the selected risk factors.

**Table 3:** Colorectal cancer mortality and incidence attributable to the selected risk factors in China, 2012

Risk factors	Men			Women			Total		
	PAF (95% CI)^a^ (%)	Mortality	Incidence	PAF (95% CI)^a^ (%)	Mortality	Incidence	PAF(95% CI)^a^ (%)	Mortality	Incidence
Tobacco smoking	8.4(4.9–12.1)	6636	12,348	0.4(0.2–0.6)	240	428	4.9(2.9–7.1)	6876	12,776
Alcohol drinking	8.7(0.4–17.6)	6873	12,789	1.1(0.0–2.4)	660	1177	5.4(0.2–11.0)	7533	13,966
Overweight/Obesity	4.9(1.6–8.5)	3871	7203	5.8(1.9–9.9)	3480	6206	5.3(1.7–9.1)	7351	13,409
Physical inactive	8.9(6.5–11.1)	7031	13,083	9.0(5.9–13.1)	5400	9630	8.9(6.1–22.2)	12,431	22,713
Low vegetable intake	18.8(6.2–29.1)	14,852	27,636	16.8(5.7–25.9)	10,080	17,976	17.9(3.7–17.1)	24,932	45,612
Low fruit intake	6.2(2.2–10.4)	4898	9114	6.7(2.3–11.2)	4020	7169	6.4(2.5–13.2)	8918	16,283
High red and processed meat intake	9.1(2.7–14.8)	7189	13,377	7.9(2.2–12.7)	4740	8453	8.6(2.5–13.9)	11,929	21,830
Total^b^	49.8(22.2–68.1)	39,342	73,206	39.6(17.1–56.4)	23,760	42,372	45.5(18.1–63.8)	63,102	115,578

*PAF* Population Attributable Fraction. Colorectal cancer incidence and mortality in China were from the Globocan database in 2012 [22]

^a^95% CIs were calculated by a delta method

^b^Combined PAF for smoking, drinking, overweight/obesity, physical inactivity, low vegetable intake, low fruit intake, high red and processed meat intake was calculated using the following formula [35]: *PAF* = 1 – (1 – *PAF*_1_) × (1 – *PAF*_2_) × (1 – *PAF*_3_) × (1 – *PAF*_4_) × (1 – *PAF*_5_) × (1 – *PAF*_6_) × (1 – *PAF*_7_)

## Discussion

Our study is for the first time to comprehensively estimate the burden of colorectal cancer incidence and mortality attributable to the known risk factors in China. Overall, we estimated that 45.5% of colorectal cancer incidence and mortality (49.8% in men and 39.6% in women, respectively) were attributable to the selected environmental risk factors in China in 2012, including tobacco smoking, alcohol drinking, overweight/obesity, physical inactivity and dietary factors. Our results showed that PAFs of smoking and alcohol drinking were 8.4% and 8.7% in men, and 0.4% and 1.1% in women, respectively. Low vegetable intake was responsible for the most incidence and mortality of colorectal cancer for both genders, with 18.8% in men and 16.8% in women.

We compared our estimates of the fraction of joint avoidable causes of colorectal cancer with similar estimates from previous studies in different countries as showed in Table 4. PAFs for colorectal cancer in our study were higher than the corresponding estimates in the worldwide study [37], French study [14], and Japan study [17], but a little lower than that in the UK study [15]. The discrepancy of PAFs in these studies could be explained by the selected risk factors, source of data on the prevalence and RR, and genetic diversity. For instance, tobacco smoking and alcohol drinking among women are much lower in China compared with other Asian (such as Japan) and European populations (such as France).

**Table 4:** Comparison of Population Attributable Fraction (PAF, %) of colorectal cancer mortality and incidence attributable of modified risk factors in various studies

Studies	Men	Women	Total	Risk factors included
Our study^a^	49.8	39.6	45.5	Tobacco smoking, alcohol drinking, overweight and obesity, physical inactivity, high red and processed meat intake, low fruit and vegetable intake
Worldwide study^a^[37]	–	–	13.0	Overweight and obesity, physical inactivity, low fruit and vegetable intake
Low and middle income countries^a^	–	–	11.0	
High income countries^a^	–	–	15.0	
French study^a^ [14]	21.3	16.0	18.8	Alcohol drinking, obesity and overweight and physical inactivity
UK study^a^ [15]	56.5	51.9	54.4	Tobacco smoking, alcohol drinking, intake of meat and fibre, overweight and obesity, Physical excises, infections and radiation-ionizing
Japan study [17]				Tobacco smoking, alcohol drinking, overweight and obesity and physical inactivity
Colon	51.0	12.8	33.6	
Rectum	46.6	6.5	31.5	

^a^PAFs for colorectal cancer

Tobacco smoking, as a cause of cancer, is common in China. Tobacco smoking was responsible for five million deaths in 2000, with 50% of these deaths in low and middle income countries. Zheng and his colleagues examined the burden of tobacco smoking-related deaths in Asia [38], which indicated that 3.1% of colorectal cancer deaths among mainland Chinese women were attributable to smoking. Reasons for the discrepancy of PAF may be explained by the different sources of prevalence and RR in these studies. In Zheng’s study, the prevalence of smoking was from a cross-sectional survey conducted in 1990s, and RR was estimated from four cohort studies in mainland China among subjects over 44 years; while in our study, the prevalence data was derived from a national survey in 2002, and RR was obtained from the combined findings in a meta-analysis. We also conducted a sensitivity analysis using the smoking prevalence in 1997 (assumed a lag-time of 15 years) to estimate PAF, and our results remained robust with respect to different lag-time.

Alcohol consumption is causally associated with the increased risk of certain cancers, including colorectal cancer. A previous study in China has evaluated the role of alcohol on the cancer burden in 2005 and showed that PAFs of colorectal cancer were 2.06% in men and 0.15% in women based on the 1991 drinking habits [19], while the corresponding PAFs were 2.32% in men and 0.27% in women based on the prevalence in 2002. The RR used in Liang’s study was extracted from a meta-analysis in China in 2003 and the value was not statistically significant. The increased trend of PAFs with years was observed in her study, which may explain the much higher PAFs in our estimates calculated based on the increased prevalence of 2002 drinking habits. A sensitivity analysis for estimating PAF using the prevalence in 1997 did not alter our results, which remained also robust with respect to different lag-time.

Few studies can be available for PAF of colorectal cancer attributable to overweight and obesity and physical inactivity in China. Only one similar study was available in China [20], and showed that overweight and obesity were responsible for 0.7% of colorectal cancer deaths for men and 1.5% of rectal cancer deaths for women in 2005, which were lower than our estimates. Our study indicated that overweight and obesity caused 4.9% of colorectal cancer deaths in men and 5.8% in women, respectively. Moreover, Wang’s study [20] estimated that 23.7% of colon cancer among urban men and 11.0% among urban women were attributable to physical inactivity. The corresponding PAFs in our study were 8.9% in men and 9.0% in women for both urban and rural areas. The difference for PAF could be explained by the source of prevalence of physical inactivity.

Many previous studies have reported the combined effect of both fruit and vegetable intake on cancer burden, but no studied can be available for the effect of red and processed meat on colorectal cancer incidence and mortality. To our knowledge, our study was for the first time to estimate the PAF for high red and processed meat intake in China. All the selected dietary factors were responsible for 31.4% of colorectal cancer incidence and mortality, which showed a quite large combined effect. However, Danaei and his colleagues found that only 2% of colorectal cancer deaths in low and middle income countries were attributable to low fruit intake and low vegetable intake [37], reflecting the different sources of RRs and exposure rates in these two studies.

The strengths of our study included first systematic assessment of colorectal cancer causes, prevalence data from nationally representative studies, and new national data on colorectal cancer incident cases and deaths. However, our results have several limitations. First, several other known risk factors of colorectal cancer exist but have not been included in our study. For example, further emerging evidence suggests that infection with *Helicobacter pylori*, Fuso bacterium species, and other potential infectious agents might be associated with an increased risk of colorectal cancer [39–41], but their causal relationships with colorectal cancer risk are unclear. Furthermore, epidemiological studies have consistently shown an inverse association between serum vitamin D concentrations and risk of colorectal cancer, but whether and to what extent this association is causal needs to be established [42]. Even the selected risk factors in our study can also be classified into more specific groups, for example, smokers can be divided into former smokers and current smokers; type of alcohol drinking can be divided into beer, spirit and others, which can also affect our estimates. Therefore, future studies are still needed to evaluate the contribution of these risk factors and others to the burden of colorectal cancer in China. Second, some sources used for PAF calculations might be uncertain. For data on prevalence, self-reported questionnaires may lead to underestimation of prevalence of exposure (such as smoking and alcohol consumption), which may underestimate the PAFs in our study. These data were extracted from different studies in China, which may also affect our PAF estimates. For relative risks used in PAF calculations, the best priority should be given to the meta-analyses included all related studies in China, but prospective cohort studies in China were used as a substitute for certain risk factors due to lack of the nation-based meta-analyses. For dietary factors, we derived RRs from a meta-analysis in the worldwide population. As a result, there could be uncertainty in the extrapolation of these RRs to the Chinese population. The direction and potential magnitude of potential bias could not be evaluated as well. Moreover, we used equal RRs for both genders when data for men and women separately were unavailable, but the RRs for men and women might be different in reality that could make our result statistical instability. Third, we did not adjust the PAFs for the potential interaction of the selected risk factors because few data from China can be available to provide the accurate RRs for interaction between these risk factors. Combined PAF for seven risk factors, however, was estimated in our study.

## Conclusions

In summary, our study represents a systematic assessment of the burden of tobacco smoking, alcohol drinking, overweight/obesity, physical inactivity, low fruit intake, low vegetable intake and high red and processed meat intake on colorectal cancer incidence and mortality in China. Approximately 46% of colorectal cancer incidence and mortality in the Chinese population were attributable to these seven risk factors in 2012. This report reinforces the notion that increasing vegetable and fruit intake and reducing red and processed meat intake are by far the main avenues for reducing the burden of colorectal cancer in China, but other risk factors, including alcohol drinking and physical inactivity, should not be neglected.

## Appendix 1

**Table 5:** Risk factors of colorectal cancer included in the study

Risk factors	Definition of theoretical minimum risk exposure
Tobacco Smoking	Never smoking
Alcohol drinking	Less than 12 occasions during the past 12 months
Overweight and Obesity	Body mass index <25 kg/m^2^
Physical inactivity	less than 30 min of moderate or vigorous activity per day
Low vegetable intake	Higher than the lowest quintile group
Low fruit intake	Higher than the lowest quintile group
High red and processed meat intake	Lower than the highest quintile group

**Table 6:** Prevalence of smoking, alcohol drinking, overweight, obesity and physical inactivity in China around 2000

Risk factors	Men (%)	Women (%)	Year	Reference
Tobacco Smoking	57.4	2.6	2002	25
Alcohol drinking	39.6	4.5	2002	26
Physical inactivity	30.4	36.8	2000	28
Overweight	15.0	17.0	2000	29
Obesity	2.5	3.4	2000	29

## Abbreviations

95% CI: 95% confidence interval
BMI: Body mass index
PAF: Population attributable fraction
RR: Relative risk
